# Downregulated hsa_circ_0077837 and hsa_circ_0004826, facilitate bladder cancer progression and predict poor prognosis for bladder cancer patients

**DOI:** 10.1002/cam4.3006

**Published:** 2020-04-06

**Authors:** Chong Shen, Zhouliang Wu, Yujie Wang, Shen Gao, La Da, Linguo Xie, Yunkai Qie, Dawei Tian, Hailong Hu

**Affiliations:** ^1^ Department of Urology The Second Hospital of Tianjin Medical University Tianjin China; ^2^ Tianjin Key Laboratory of Urology Tianjin Institute of Urology The Second Hospital of Tianjin Medical University Tianjin China

**Keywords:** bioinformatic analysis, Bladder cancer, circular RNAs, high‐throughput RNA sequencing, invasion, prognosis

## Abstract

Growing evidence has indicated that circular RNAs (circRNAs) play crucial roles in multiple biological processes. However, alterations in circRNA profiles during bladder cancer progression and the clinical significance thereof remain unclear. Therefore, high‐throughput RNA sequencing was conducted to identify circRNA and mRNA profiles in five pairs of bladder cancer tissues and adjacent noncancerous tissues. A total of 87 differentially expressed circRNAs and 2756 mRNAs were detected in above bladder cancer samples compared with paired noncancerous samples. Functional enrichment analyses, circRNA‐microRNA‐mRNA, and protein‐protein interaction networks revealed that these dysregulated circRNAs were potentially involved in carcinogenesis and evolution of bladder cancer. Subsequently, the differential expression of eight circRNAs was detected by real‐time qPCR. Hsa_circ_0003141 and hsa_circ_0008039 were significantly upregulated as well as hsa_circ_0026782, hsa_circ_0077837, hsa_circ_0004826, and hsa_circ_0001946 were significantly downregulated among validation of 70 matched bladder cancer tissues (≥75%). Moreover, hsa_circ_0077837 and hsa_circ_0004826 were also verified as markedly downregulated in four bladder cancer cells (100%). Naturally, hsa_circ_0077837 and hsa_circ_0004826 were also demonstrated using RNase‐R+ resistance experiments. In addition, Fisherʹs exact test, Kaplan‐Meier plots, Cox regression analyses, and receiver operating characteristic curve was performed to assess their clinical value. Downregulation of hsa_circ_0077837 and hsa_circ_0004826 all was significantly correlated with worse clinicopathological features and poor prognosis of bladder cancer patients. The area under the receiver operating characteristic curve of them was 0.775 (*P* < .0001) and 0.790 (*P* < .0001), respectively. Not surprisingly, in vitro functional experiments also demonstrated that the overexpression of hsa_circ_0077837 and hsa_circ_0004826 significantly weakened the proliferation, migration, and invasion of bladder cancer cells. Overall, hsa_circ_0077837 and hsa_circ_0004826 might act as tumor suppressors in the bladder cancer progression and serve as a potential biomarker for the diagnosis, prognosis, and therapy of bladder cancer.

## Graphical abstract

The transcriptional data of circRNAs and mRNAs were deeply detected by high‐throughput RNA sequencing and systematically analyzed using bioinformatics in patients with BC; and these eight dysregulated circRNAs were also verified by real‐time qRT‐PCR. Correlation analysis of clinicopathological features and survival analyses of circRNAs in BC were conducted. Functional experiments with circ_0077837 and circ_0004826 overexpression significantly weakened the progression of BC cells.

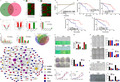

## 1 BACKGROUND

Bladder cancer (BC) belongs to the most commonly occurring malignancies of the urinary system and the ninth most frequently diagnosed cancer type with both high incidence and mortality rates worldwide.^1^, ^2^ Based on the depth of tumor infiltration, BC is categorized into muscle‐invasive bladder cancer (MIBC) (Ta, Tis, and T1, approximately accounting for 70%‐75%) and nonmuscle invasive bladder cancer (NMIBC) (T3‐T4, approximately 25%‐30%).^3^, ^4^, ^5^ Of the patients with NMIBC, it has a higher recurrence rate after local tumor resection and is very likely to progress to MIBC.^6^ For the patients with MIBC, distant metastases occur in the lungs, bones, and liver more frequently, and are closely related to poor prognosis.^7^, ^8^ Up to now, no effective therapies are available for BC patients with tumor recurrence or metastasis.^9^ Therefore, it is of great clinical value to illuminate the molecular mechanisms that drive the tumorigenesis and progression of BC, which will contribute in developing more effective anticancer therapies.

Circular RNAs (circRNAs), are a subclass of noncoding RNAs that lack neither 5ʹ caps nor 3ʹ polyadenylated tails, covalently form closed continuous loops and thereby have more stability than their linear types.^10^, ^11^ Differed from linear RNAs that are formed by classical splicing, circRNAs, formed through back‐splicing of pre‐mRNA transcripts, are highly rich, conserved, stable, and tissue/developmental‐stage/disease specific in the eukaryotic transcriptomes,^12^, ^13^, ^14^ but their the characterizations and functions remain largely elusive for many years.

Recent evidences have suggested that circRNAs involve in far‐ranging biological processes, including transcription, mRNA splicing, RNA decay and translation, and their deregulation takes pivotal roles in the development and metastasis of various tumors, such as glioma,^15^ hepatoblastoma,^16^ colorectal cancer,^17^ lung cancer,^18^ prostate cancer,^19^ bladder cancer,^20^ and gastric cancer.^21^ For example, Li et al revealed that circANKS1B promotes colorectal cancer cell migration and invasion by working as a molecular sponge of miR‐149 to regulate FOXM1 and Slug protein expressions.^17^ It has been showed that hsa_circ_0007059 abates cell proliferation and EMT progress in lung cancer cells through inactivation of Wnt/β‐catenin and ERK1/2 pathways via inhibiting miR‐378.^18^ In BC, Liu et al demonstrated that circDOCK1 promotes BC progression through modulation of circDOCK1/hsa‐miR‐132‐3p/Sox5 pathway in vitro and in vivo as well as acts as a promising biomarker and therapeutic targets for BC.^20^ And notably, our preliminary works have illustrated that high expression of circASXL1 in BC connects with poor TNM classification and may independently predict overall survival (OS) for patients with BC.^22^ These findings strongly supported the idea that circRNAs may be intimately associated with the pathogenesis, used as potential attractive diagnosis and prognosis biomarkers and therapeutic target for diverse cancer. In the bladder cancer, although there have been some reports on circular RNA, such as circ‐ITCH and circACVR2A suppresses bladder cancer progression by miR‐17/miR‐224/p21/PTEN and miR‐626/EYA4 axis, respectively^23^, ^24^; however, the global expression profile and precise molecular mechanism of BC‐specific circRNAs has not been well uncovered.

Therefore, using high‐throughput RNA sequencing (RNA‐seq), we here investigated the expression profiles of circRNAs, mRNAs and identified 87 remarkably differentially expressed (DE) circRNAs, 2756 significantly dysregulated mRNAs in BC tissues compared with adjacent noncancerous tissues. Then, we performed Gene Ontology (GO) analysis and Kyoto Encyclopedia of Gene and Genome (KEGG) pathway analysis of the host genes of circRNAs.^25^, ^26^ Subsequently, six circRNAs were selected based on the difficulty degree of circRNA‐specific primer design and their parental genesʹ relevance to cancer. Their expression levels were examined by quantitative real‐time polymerase chain reaction (qRT‐PCR) in both 70 BC specimens and 4 BC cells, and the results verified the RNA‐seq data.

According to the validation results, both circ_0077837 and circ_0004826 was decreased in BC tissues and associated with unfavorable clinicopathological parameters and poor survival prognosis of BC patients. Additionally, to explore the latent molecular mechanism of circRNAs in patients with BC, we predicted circRNAs‐targeted the first five miRNAs, miRNAs‐targeted crucial mRNAs intersecting with RNA‐seq data by famous network database and constructed circRNAs‐related competitive endogenous RNA (ceRNA) network. For these downstream intersecting DE genes, we also performed GO function, KEGG pathway and protein‐protein interaction (PPI) networks analysis. Furthermore, in vitro experiments demonstrated that the overexpression of circ_0077837 and circ_0004826 could inhibit proliferation, migration, and invasion of BC cells. Therefore, both circ_0077837 and circ_0004826 can act as a promising biomarker for diagnosis and prognosis predication and as a potential target in BC therapy. In summary, our study expounded that circRNAs are dysregulated in BC and play crucial effects in the aggressiveness behaviors of BC cells.

## 2 METHODS

### 2.1 Tissue specimens

This study recruited 47 patients with BC undergoing Radical cystectomy and 23 ones conducting TURBT surgery between September 2015 and August 2019 in the urology department of Second Affiliated Hospital, Tianjin Medical University (China). All recruited patients were newly confirmed by histopathology and thereof treated and did not receive preoperative chemotherapy, radiotherapy, or other therapies (Detailed data of these BC patients are supplied in Table S1). Among them, five pairs of tumor tissues and adjacent normal tissues were used for the high‐throughput RNA sequencing. Moreover, a written informed consent was acquired from every patient or their family. This study was approved by the Institutional Review Board in this hospital.

### 2.2 Cell culture

The human BC cell lines (EJ, 5637 and T24) and human immortalized uroepithelial cell line SV‐HUC‐1 was obtained from the Chinese Academy of Sciences Cellbank. The BC cell line 253J‐BV, highly tumorigenicity and metastasis, were developed from 253J cells^27^, ^28^ and kindly provided by Professor Lei Li (The First Affiliated Hospital of Xiʹan Jiaotong University, Xiʹan, China). These BC cells were maintained in the RPMI‐1640 (BI Company) medium, supplemented with 10% fetal bovine serum (FBS; Gibco) and 1% penicillin‐streptomycin (Gibco) in a humidified incubator containing with 5% CO_2_ at 37℃. Human uroepithelial SV‐HUC‐1 cell were cultured in the F‐12k Nutrient Mixture (Gibco) with above identical material condition under the same culture atmosphere.

### 2.3 Cell transfection

For lentivirus infection, the circ_0077837 and circ_0004826 overexpression (OV‐) lentivirus and the negatively control (NC) (Hanbio Biotechnology Co.) were, respectively, alone transduced into EJ and 253J‐BV cell lines as per manufacturerʹs protocols. Infection efficiency was determined through counting the GFP‐positive cells under a inverted fluorescence microscope (Olympus IX70) at 72 hours after infection, and the overexpression efficiency of objective circRNAs was evaluated by quantitative real‐time polymerase chain reaction (qRT‐PCR) analysis.

### 2.4 Total RNA isolation and quality control (QC)

Total RNA was isolated from surgical tissue samples stored in −80℃ refrigerator and the cultured cells using TRIzol reagent (Invitrogen) according to the manufacturerʹs protocol. The concentrations of extracted RNA were detected applying a NanoDrop ND‐1000 (Thermo Fisher Scientific). In this study, all RNA samples passed the quality standard based on a qualified ratio of OD260 to OD280 (1.8‐2.1). The RNAʹs integrity and gDNA contamination test for high‐throughput sequencing analysis was estimated through conducting denaturing agarose gel electrophoresis (Figure S1).

### 2.5 RNA‐seq analysis

Initially, ribosomal RNA (rRNA) was depleted from total RNA utilizing the Ribo‐Zero rRNA Removal Kit (Illumina) conforming to the manufacturerʹs protocols. The rRNA‐removed RNA was used to construct the RNA‐seq libraries with TruSeq Stranded Total RNA Library Prep Kit (Illumina) following the manufacturerʹs instructions. RNA quality and quantity in the libraries was controlled using the BioAnalyzer 2100 system (Agilent Technologies). The RNA libraries were denatured as single‐stranded DNA molecules, captured on Illumina Flow Cells (Illumina), amplified in situ as clusters and then sequenced for 150 cycles on Illumina HiSeqTM 4000 Sequencer (Illumina) according to the manufacturerʹs protocols.

### 2.6 CircRNA profiling analysis

Briefly, paired‐end reads were gained from Illumina HiSeqTM 4000 sequencer (Illumina). After 3ʹ adaptor‐trimming and low‐quality reads were cut out by the cutadapt software (v1.9.3), the high‐quality reads were aligned to the reference genome using STAR software (v2.5.1b).^29^ Next, circRNAs were detected and annotated with the DCC software and the circBase database, respectively. The edgeR software (v3.16.5) was employed to normalized the data and performed for differentially expressed circRNA analysis.^30^

### 2.7 Bioinformatics analysis

CircRNAs have been shown to function as regulators of host gene transcription and alternative splicing and miRNA sponges.^31^ Accordingly, GO and KEGG pathway analyses were conducted for the host genes and intersecting target genes of differentially expressed circRNAs using the DAVID (Database for Annotation, Visualization, and Integrated Discovery) Bioinformatics resources (http://david.abcc.ncifcrf.gov/).^32^ Among them, GO analysis can be classified into biological process (BP), cellular component (CC), and molecular function (MF).

### 2.8 Analyses of circRNA‐miRNA‐mRNA (ceRNA) and protein‐protein interaction (PPI) interactions in BC

For detecting the interactions between mRNAs and circRNAs from sequencing result, the circRNAs‐miRNAs‐mRNAs ceRNA network was established. The circRNA‐miRNA potential correlation was predicted by miRNA target prediction software based on circular RNA Interactome (https://circinteractome.nia.nih.gov/index.html)^33^ and MiRanda (http://www.microrna.org). Analogously, MiRTarBase (http://mirtarbase.mbc.nctu.edu.tw/)^34^ and miRDB (http://www.mirdb.org/index.html)^35^ were used for predicting the miRNA‐binding mRNAs. To get a better understanding of these DE‐circRNAs in circRNA‐miRNA‐mRNA interaction network, the construction and visualization of the ceRNA network was accomplished by Cytoscape 3.6.1. Afterward, according to 94 intersection target DE genes of screened eight DE‐circRNAs, the PPI network was performed using STRING (Version 11.0) (https://string‐db.org/) online analysis and Molecular Complex Detection (MCODE) were utilized to PPI network to pick out meaningful DE genes modules.

### 2.9 Reverse transcription PCR analysis, RNase‐R treatment, and quantitative PCR analysis

Among the aberrantly expressed circRNAs identified, eight circRNAs were selected for validations using qRT‐PCR in 70 newly diagnosed BC patients. Subsequently, total RNAs extracted from tumorous and paired adjacent normal tissues from these patients were utilized to reverse transcription using cDNA synthesis kit (Roche Diagnostic Co.). Total RNA was treated with RNase‐R+ (Epicentre) previously to cDNA synthesis to detect resistance of circRNA to RNase‐R digestion. Quantitative PCR was performed using FastStart Universal SYBR Green Master Mix with ROX (Roche) on the ABI 7900HT fast real‐time PCR system (Applied Biosystems). The primers for circRNAs were synthesized by Sango Biotech. The primer sequences are shown in Table 1. GAPDH was served as an internal control gene.

**TABLE 1:** The primers used for real‐time PCR are designed and synthesized by Sango Biotech (Shanghai, China) as well as shown in Table 1

Gene name	Primer type	Primer sequence	Product length
hsa_circ_0003141	Forward primer	5ʹ‐ACAATCAGATGGCACCAGGG‐3ʹ	81
	Reverse primer	5ʹ‐CTGCCACGTCCAAATCCAGG‐3ʹ	
hsa_circ_0008039	Forward primer	5ʹ‐CTTCGCTCACCTGGATGACAA‐3ʹ	115
	Reverse primer	5ʹ‐GTACAGCTCACAGCCCTTCAG‐3	
hsa_circ_0001346	Forward primer	5ʹ‐AGTGGGCATCTGTCTCATCT‐3ʹ	151
	Reverse primer	5ʹ‐GCATCTCGTTGTAAAATCACCTT‐3ʹ	
hsa_circ_0026782	Forward primer	5ʹ‐CCTTCTCCCCTGATAGCCAC‐3ʹ	86
	Reverse primer	5ʹ‐CTTGCCTCATATCGGTGTGC‐3ʹ	
hsa_circ_0077837	Forward primer	5ʹ‐CCTGGAGAAACATGCCAAGGG‐3ʹ	160
	Reverse primer	5ʹ‐TCACTTCAGACACAGAGCCTACT‐3ʹ	
hsa_circ_0004826	Forward primer	5ʹ‐CAGCTGACTTCCCTGAAGGT‐3ʹ	111
	Reverse primer	5ʹ‐AGTCTGGCCAAGCTCTCGAA‐3ʹ	
hsa_circ_0030586	Forward primer	5ʹ‐TGGAGGAGGAAATGTAACCGAG‐3ʹ	223
	Reverse primer	5ʹ‐GTTGGGGTCTCTGATGCCTATT‐3ʹ	
hsa_circ_0001946	Forward primer	5ʹ‐TTCCAACGTCTCCAGTGTGCT‐3ʹ	126
	Reverse primer	5ʹ‐ACTTGAAGTCGCTGGAAGACCC‐3ʹ	
Gapdh	Forward primer	5ʹ‐CGGAGTCAACGGATTTGGTC‐3ʹ	180
	Reverse primer	5ʹ‐TTCCCGTTCTCAGCCTTGAC‐3ʹ	

### 2.10 Cell counting kit‐8 (CCK‐8) and clone formation assays

For CCK‐8 assay, 0.2 × 10^4^ BC cells were seeded into the 96‐well plate and then culture for 24, 48, 72, and 96 hours in the incubator (Thermo Fisher Scientific) at 37°C equipped with 5% CO2. Subsequently, each well was supplemented with 10 μL CCK‐8 solution (Boster Bio) and incubated for 3.5 hours in dark. The absorbance at 450 nm was detected by a microplate reader (VersaMax Microplate reader). For clone formation assay, 0.5 × 103 BC cells were plated into 6‐well plates and cultured for 1 week. Colonies with diameter greater than 1 mm were then fixed with 4% paraformaldehyde for 15 minutes, stained for 15 minutes with 0.1% crystal violet (Solarbio) and counted using ImageJ software.

### 2.11 Wound‐healing experiment

Transfected BC cells were cultured in 6‐well plates and scraped using 200 μL tips after reaching approximately 90%‐95% confluency. Cells were washed thrice by PBS (Gibco) to remove floated cells and debris. Next, width of the scratch gap was photographed by an inverted microscope (Olympus) at 0 and 36 hours

### 2.12 Transwell migration and invasion assays

The cell migration and invasion assays were conducted using transwell chambers (0.8 µm; Corning) with (for invasion assays) or without (for migration assays) the Matrigel (Corning) according to the manufacturerʹs instructions. Approximately 4 × 104 cells were seeded in upper well with 200 μL serum‐free medium. The 700 μL culture medium containing 20% FBS (Gibco) was placed in the lower chamber.

After 36‐hour incubation, cells at the bottom of the membrane were fixed with 4% paraformaldehyde (Sigma) for 15 minutes and stained with 0.1% crystal violet (Solarbio) for 15 minutes at room temperature. The stained cells were counted and imaged in five randomly selected fields under an olympus microscope (Olympus). Detection was repeated three times in duplicate.

### 2.13 Statistical analysis

Statistical analysis was performed using GraphPad Prism 7.0 and the Statistical Product and Service Solutions (SPSS) 20.0 software (SPSS). The differences between BC tissues and paired normal tissues were analyzed by paired t‐test. Fisherʹs exact test was used to test the association between two categorical variables. The (ROC) curve and Kaplan‐Meier plot were constructed to evaluate the diagnostic and prognostic values, respectively. Survival data were further estimated using the univariate and multivariate Cox proportional hazards model. A *P* < 0.05 was considered statistically significant.

## 3 RESULTS

### 3.1 CircRNA and mRNA expression profiles in BC

**FIGURE 1:**
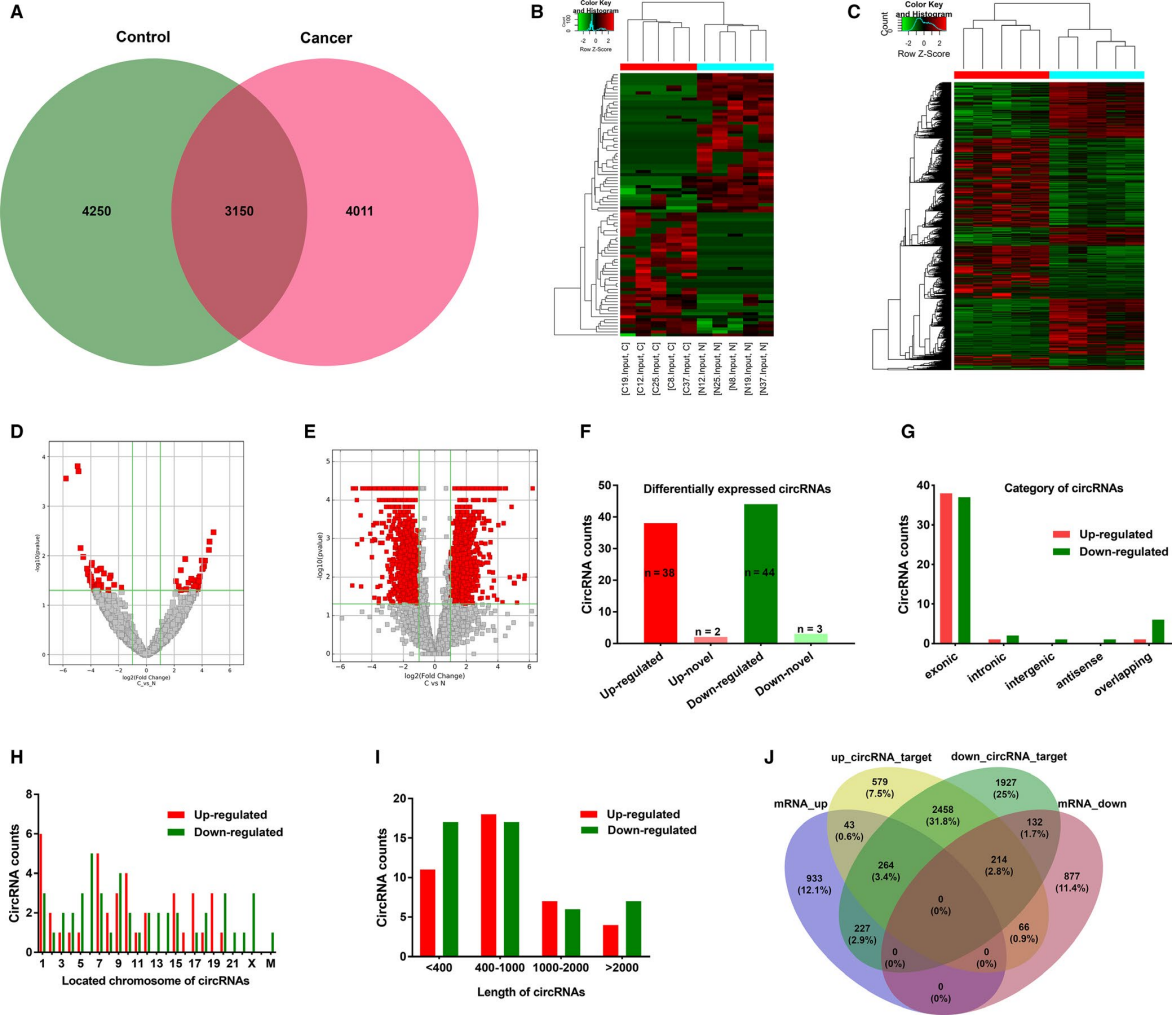
The Profiling and Characteristics of circRNAs and mRNAs in BC tissues vs adjacent nontumor tissues. (A), Venn diagram demonstrated the number of overlapping circRNAs between the BC (marked as “Cancer”) and adjacent nontumor tissues (“Control”). (B and C) Heatmap for the 87 significantly differentially expressed (DE) circRNAs and 2756 DE mRNAs in the five matched BC specimens. Each column represents a sample and each row corresponds to a transcript. Dendrograms produced by clustering analysis of the samples and transcripts are displayed on the top and left, respectively. Red indicates upregulation and green indicates downregulation. DE: Fold Change ≥2 (or −2); *P* < .05. “C”, cancer tissues. “N”, adjacent normal tissues. (D and E) Volcano plot for these DE circRNAs and mRNAs in the five paired BC samples. Red squares show the DE circRNAs and mRNAs in BC tissue samples compared with the adjacent tissue samples. (F) Among DE circRNAs, 40 circRNAs were significantly upregulated (red), and of which, two were novel (light red). 47 circRNAs were significantly downregulated (green), and of which, three were novel (light green). (G) The counts of DE circRNAs based on their categories of circle components. (H) The distribution of DE circRNAs based on the location on human chromosomes. “M” represents mitochondrial genome. (I) The nucleotide length distribution of these DE circRNAs. J, Venn diagram displayed overlapping number between predicted targeted mRNAs of DE circRNAs and our sequencing mRNAs result

First, we analyzed the profiling of circRNAs and mRNAs in cancer tissues and paired noncancer tissues from five BC patients by RNA‐seq. 11 411 circRNA transcripts in total were identified in both BC and non‐BC tissues, including 5578 upregulated circRNAs and 5833 downregulated circRNAs in BC tissues vs non‐BC tissues. For these circRNAs, 4250 were only detected in the paired control tissues, 4011 were only identified in BC tissues and 3150 were identified in both groups (Figure 1A). Similarly, a total of 20 266 mRNAs transcripts were detected, including 12 856 upregulated and 7409 downregulated mRNAs. To determine the differentially expressed circRNAs, we screened those with fold change more than 2 (FC > 2) and *P* value lower than 0.05 (*P* < .05), and then identified that 40 circRNAs were remarkably upregulated, whereas 47 were significantly downregulated in the tumors compared with the controls (Table S2). Alike, we identified that 1467 mRNAs were significantly upregulated and 1289 were remarkably downregulated in BC tissues. Hierarchical clustering and volcano plots revealed that the expression profiles of circRNAs and mRNAs were apparently distinguished and clustered between the two groups of samples (Figure 1B‐E).

Among the 87 aberrantly expressed circRNAs, five circRNAs were first identified as novel circRNAs, 82 circRNAs were detected previously and listed in the published circRNA database or articles (Figure 1F). Most differentially expressed circRNAs originated from exons (Figure 1G). The circRNAs are distributed on most of human chromosomes, including 21 autosomes and the X chromosome. There was also one downregulated circRNAs located on the mitochondria (Figure 1H). The majority had a length with less than 2000 nucleotides (nt) (Figure 1I). Venn diagram exhibited the cross‐gene number between predicted targeted mRNAs of differentially expressed circRNAs and our sequencing mRNAs result (Figure 1J).

### 3.2 GO and KEGG pathway analysis of the parental genes and crucial target genes of differentially expressed circRNAs

**FIGURE 2:**
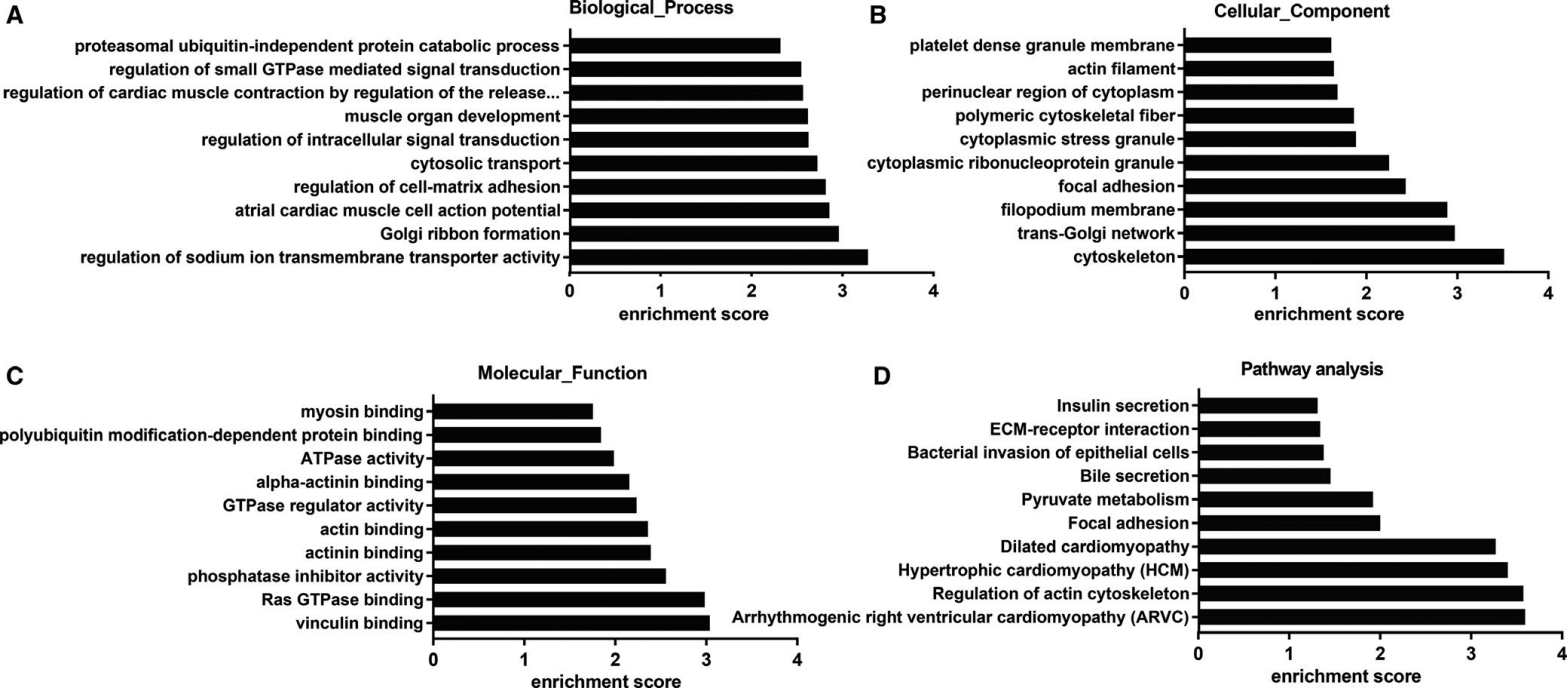
GO annotation and KEGG Pathways analyses of DE‐circRNAsʹ parental genes. A, B and C, GO analysis was conducted to obtain three domains, including biological process, cellular component and molecular function. D, KEGG pathway enrichment analysis of these parental genes. GO, Gene Ontology; KEGG, Kyoto Encyclopedia of Genes and Genomes; DE, differentially expressed

Increasing studies have reported that some circRNAs play crucial role in modulating the expression of their host genes.^36^, ^37^ Therefore, we performed GO and KEGG pathway analysis of the parental genes of abnormally expressed circRNAs to speculate circRNA biological functions. GO includes three domains (biological process [BP], cell component [CC], and molecular function [MF]). The top 10 enriched GO terms in the BP, CC, and MF are shown in Figure 2. Of the BP, the predominantly enriched GO term was “regulation of sodium ion transmembrane transporter activity” (Figure 2A). The most significantly enriched GO item of the CC was “cytoskeleton” (Figure 2B). In the MF, the two main GO term was “vinculin binding” and “Ras GTPase binding” (Figure 2C). Next, we also conducted KEGG analyses of the linear counterparts that generated DE circRNAs. The top 10 enriched pathways are displayed in Figure 2D; and among them, the two most abundant terms were “Arrhythmogenic right ventricular cardiomyopathy (ARVC)” and “Regulation of actin cytoskeleton”.

**FIGURE 3:**
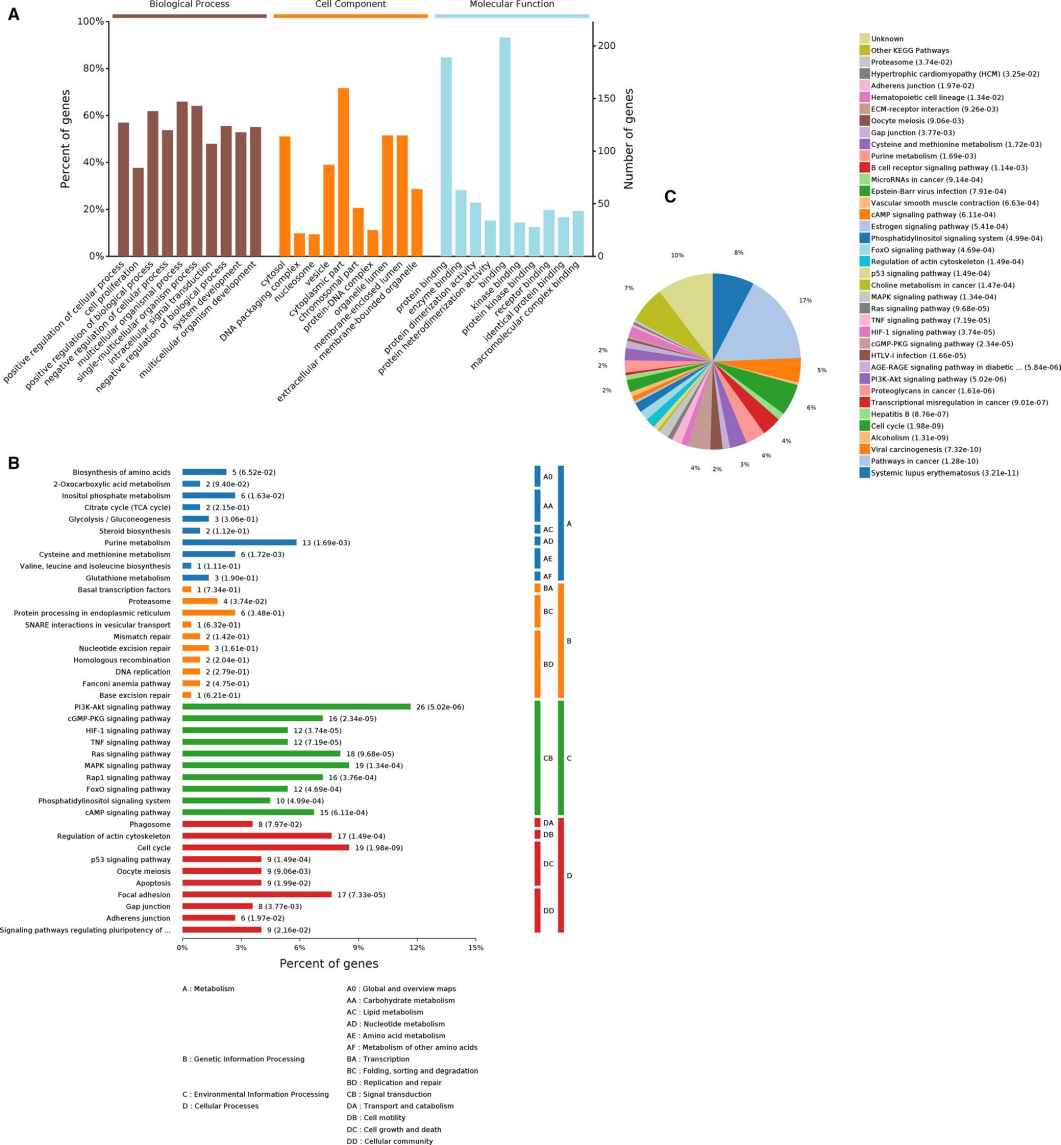
GO and KEGG pathway analyses for the intersecting target DE genes. (A) GO annotation with the top 10 enrichment scores, covering the aspects of biological process, cellular component, and molecular function, respectively. (B) The relevant significantly enriched pathways were determined for these DEmRNAs. And pathways are shown according to the following subcategories: Metabolism, Genetic Information Processing, Environmental Information Processing, Cellular Processes. (C) A pie graph is distinguished into “slices” percentage corresponding to the kegg categories to the exhibited. GO, Gene Ontology; KEGG, Kyoto Encyclopedia of Genes and Genomes; DE, differentially expressed

For the overlapping 227 key DE‐genes in Table S3, we also performed GO and KEGG pathway analysis. In the GO terms, top 10 enriched BP with brown color, CC with orange, and MF with light blue were displayed (Figure 3A). Accordingly, these genes might be most associated with “positive regulation of cell proliferation” in the BP classification. Of the CC category, “cytosol,” were the most prominent. For the MF, the most significantly enriched GO term was “protein binding.” The remarkably enriched pathways related to these DEmRNAs are presented in Figure 3B,C; and of them, the two most enriched terms were “systemic lupus erythematosus” and “pathways in cancer.”

### 3.3 Validation of dysregulated circRNAs in BC tissues and cell lines

**FIGURE 4:**
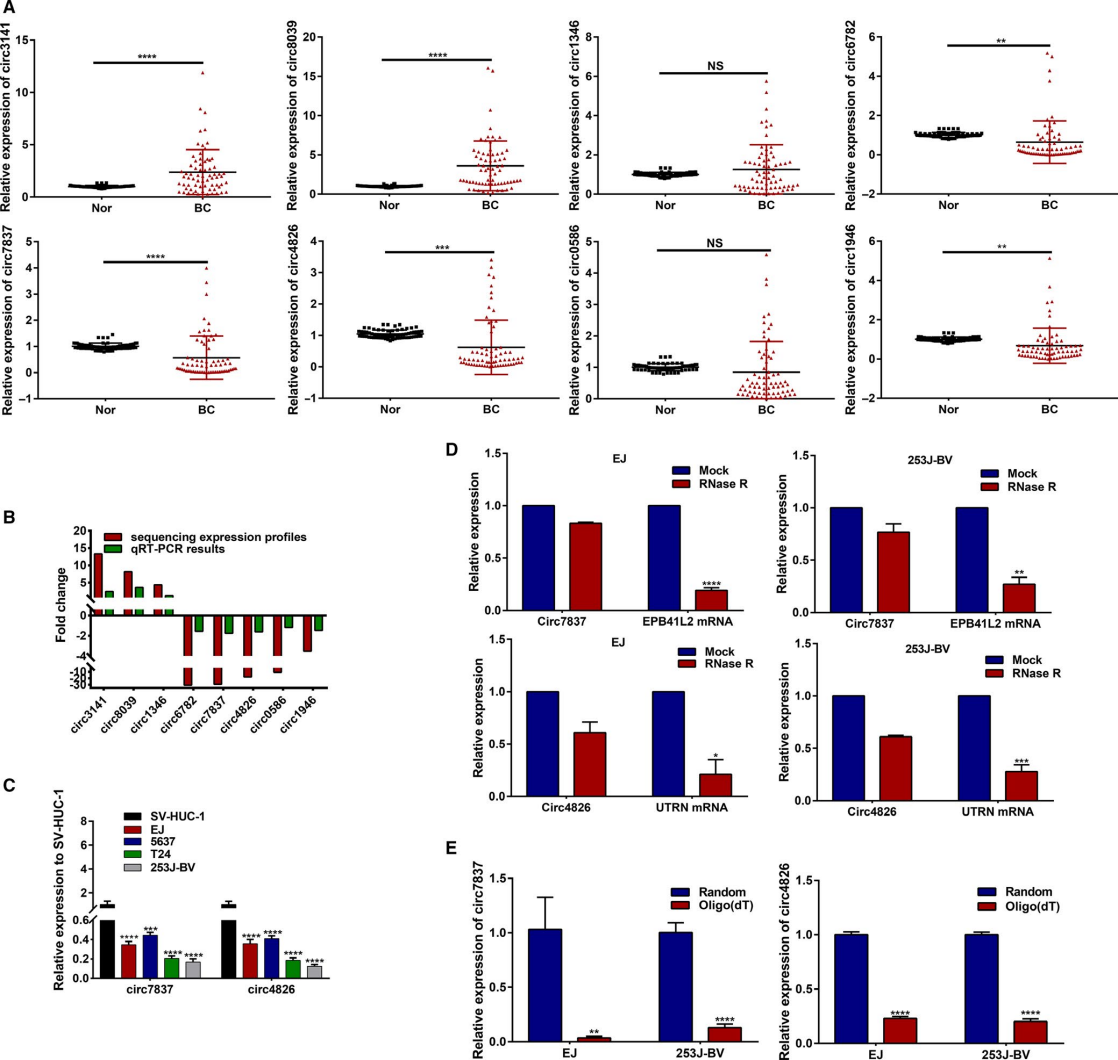
The relative levels of the selected eight circRNAs by real‐time qPCR (in triplicate) analysis. (A) Relative expression levels of them were determined in 70 pairs of BC specimens and nontumorous specimens. Circ‐3141, hsa_circ_0003141; Circ‐8039, hsa_circ_0008039; Circ‐6782, hsa_circ_0026782; Circ‐7837, hsa_circ_0077837; Circ‐4826, hsa_circ_0004826; Circ‐1946, hsa_circ_0001946. (B) Comparison of fold change (2^−△△Ct^) of circRNAs between qRT‐PCR results (n = 70 paired tissues) and our sequencing expression profiles (n = 5 paired tissues). (C) qRT‐PCR measurement of the expression levels of circ_0077837 and circ_0004826 among normal urothelial cell line (SV‐HUC‐1) and four malignant BC cell lines (EJ, HTB‐9, T24T, and 253J‐BV). (D) qRT‐PCR for the expression of circ_0077837, circ_0004826 and relevant EPB41L2, UTRN mRNA in EJ and 253J‐BV cells treated with or without RNase R. The results indicated that circ_0077837 and circ_0004826 was resistant to RNase R digestion. (E) Oligo (dT)18 primers or random hexamer were carried out in the reverse transcription experiments, respectively. For circ_0077837 and circ_0004826, the relative expression levels were analyzed by qRT‐PCR and normalized to the value through random hexamer primers

To verify our sequencing data, we randomly selected eight circRNAs based on the following standards: (1) the host gene of DE‐circRNAs was closely associated with pathogenesis and progression of various cancer. (2) the difficulty degree of circRNA‐specific primer design. Furthermore, screened circRNAs expression levels was validated in 70 paired nontumorous and tumor specimens as well as four BC cell lines with different degrees of malignancy and normal cell line by qRT‐PCR. As shown in Figure 4A,B, the circ_0026782, circ_0077837, circ_0004826, and circ_0001946 were dramatically decreased, whereas as well as circ_0003141 and circ_0008039 was significantly increased in BC tissues vs Paired noncancerous tissues, which were consistent with previous sequencing results (*P < .05, **P < .01, ***P < .001,****P < .0001). Moreover, BC cell lines with higher metastatic potential expressed a lower level of circ_0077837 and circ_0004826 (Figure 4C). Additionally, we conducted RNase‐R + digestion on these two circRNAs and examined the resistance of circRNAs to RNase‐R + digestion by RT‐qPCR. The results indicated that these two tested circRNAs all showed varying degrees of resistance to RNase‐R+ digestion compared to corresponding linear mRNAs (Figure 4D). For circ_0077837 and circ_0004826, Oligo (dT)18 primer or random hexamer were performed in the reverse transcription experiments, respectively. Subsequently, the relative expression levels were analyzed by RT‐qPCR and normalized to the value through random hexamer primers. The results showed that these two circRNAs could not been generated by oligo (dT)18 primer relative to random hexamer (Figure 4E).

### 3.4 Prediction for the circRNA‐miRNA‐mRNA and PPI interaction, and network visualization

Recent studies have reported that circRNAs bind cancer‐associated miRNAs and then affect tumor‐related genes.^38^ To explore the molecular mechanism and functions of circRNAs, we predicted potential circRNA‐binding the first five cancer‐related miRNA and thereof investigated miRNA‐binding crossed mRNAs (Table S3) using the popular prediction tools. Subsequently, the total 87 dysregulated circRNAs with 227 related targeted mRNAs were employed to establish a circRNA‐miRNA‐mRNA network by Cytoscape 3.6.1 (Figure S2). In the complicated network, one circRNA can interact with different miRNAs and one miRNA can degrade multiple mRNAs.

**FIGURE 5:**
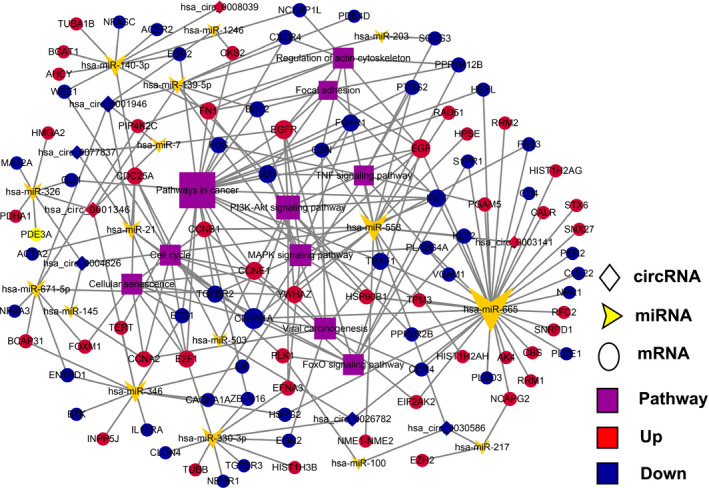
A magnified partial circRNA‐miRNA‐mRNA (ceRNAs) network for the selected eight circRNAs with 94 overlapping target genes was constructed using Cytoscape 3.6.1 tool. Of them, all of circRNAs were verified by real‐time qPCR. The yellow arrowheads, purple rectangles indicate miRNAs and pathways, respectively. The circles represent coding genes, diamonds represent circRNAs, red indicates upregulation, and blue indicates downregulation

**FIGURE 6:**
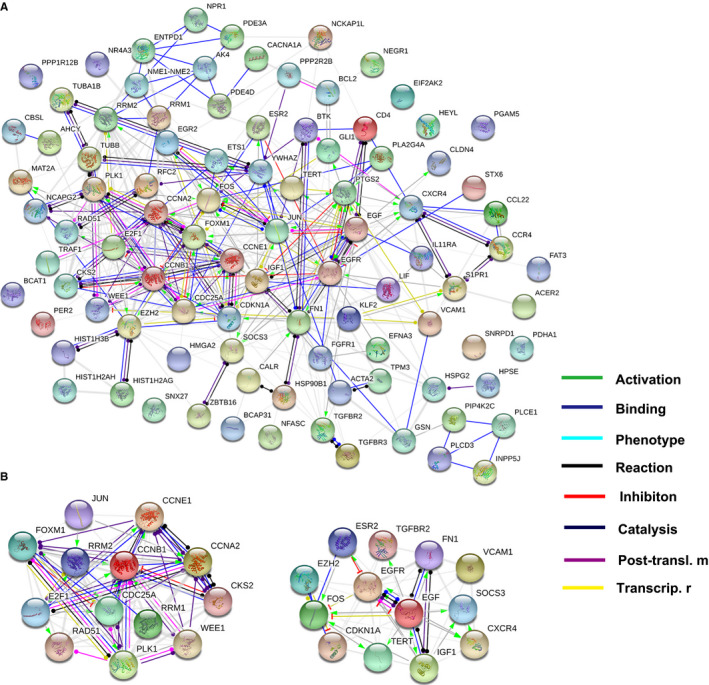
Protein‐protein interaction (PPI) network and module analysis. (A) PPI network of 94 overlapping target genes was established. (B) The top two significant modules selected from PPI network. The edges represent the interaction relationship between nodes. Posttransl. m, posttranslational modification; Transcrip. r, transcriptional regulation

Then, the selected eight DE‐circRNAs, corresponding the first five cancer‐related miRNAs for each and the total 94 hub mRNAs were chosen. The predicted miRNA response elements (MREs) for the seven key circRNAs are listed in Table 2. Ultimately, a magnified partial ceRNAs network was constructed, and presented in Figure 5. Among them, the 2D structures and binding sites of circ_0004826, hsa‐miR‐326, hsa‐miR‐558, hsa‐miR‐145, hsa‐miR‐330‐5p, and hsa‐miR‐1282 are illustrated in Figure S3A. Of circ_0077837, hsa‐miR‐7, hsa‐miR‐21, hsa‐miR‐1205, hsa‐miR‐942, and hsa‐miR‐1236, are disclosed in Figure S3B. In the network, circ_0001946 was involved in the most pairs, which was significantly downregulated in the BC samples compared with their adjacent tissue samples. Protein‐protein interaction (PPI) network of these 94 genes is shown in Figure 6A. The first two significant genes modules were screened and identified in the PPI network (Figure 6B).

**TABLE 2:** The first five cancer‐related miRNAs identification for each of the selected eight DEcircRNAs

circRNA	MRE1	MRE2	MRE3	MRE4	MRE5
hsa_circ_0003141	hsa‐miR‐1236	hsa‐miR‐758	hsa‐miR‐589	hsa‐miR‐558	hsa‐miR‐589
hsa_circ_0008039	hsa‐miR‐432	hsa‐miR‐766	hsa‐miR‐1287	hsa‐miR‐1273	hsa‐miR‐140‐3p
hsa_circ_0001346	hsa‐miR‐346	hsa‐miR‐7	hsa‐miR‐326	hsa‐miR‐139‐5p	hsa‐miR‐382
hsa_circ_0026782	hsa‐miR‐330‐3p	hsa‐miR‐503	hsa‐miR‐665	hsa‐miR‐766	hsa‐miR‐874
hsa_circ_0077837	hsa‐miR‐1236	hsa‐miR‐21	hsa‐miR‐1205	hsa‐miR‐7	hsa‐miR‐942
hsa_circ_0004826	hsa‐miR‐145	hsa‐miR‐1282	hsa‐miR‐326	hsa‐miR‐558	hsa‐miR‐330‐5p
hsa_circ_0030586	hsa‐miR‐942	hsa‐miR‐558	hsa‐miR‐217	hsa‐miR‐100	hsa‐miR‐99a
hsa_circ_0001946	hsa‐miR‐671‐5p	hsa‐miR‐203	hsa‐miR‐7	hsa‐miR‐21	hsa‐miR‐1246

Abbreviations: MRE, miRNA response element.

### 3.5 Clinical implication of selected circRNAs in BC patients

Within 70 BC tissues, these circRNAs displayed diverse expression levels despite differential expression compared with paired normal tissues, and then consistency with previous sequencing data. Therefore, the correlation between circRNAs and various clinicopathological factors of BC was assessed in the 70 patients with BC. Using “one” expression value of circRNAs as the cut‐off point for the Fisherʹs exact test and medium expression value for Kaplan‐Meierʹs survival analysis, it was found that the low level of circ_0077837, circ_0004826, and circ_0001946 expression was obviously associated with tumor invasion depth, lymph node metastasis, and higher histologic grade, particularly the first two. Whereas, the low level of circ_0026782 expression was only correlated with a lymphatic metastasis (*P* = .0322) (Table 3).

**TABLE 3:** The association analysis between expression of the screened circRNAs and clinicopathological factors of patients with BC

Variables	Circ‐3141 Exp.		Circ‐8039 Exp.		Circ‐6782 Exp.		Circ‐7837 Exp.		Circ‐4826 Exp.		Circ‐1946 Exp.	
	Low (n = 18)	High (n = 52)	Low (n = 10)	High (n = 60)	Low (n = 57)	High (n = 13)	Low (n = 54)	High (n = 16)	Low (n = 55)	High (n = 15)	Low (n = 59)	High (n = 11)
Gender												
Male (n = 61)	16	45	8	53	51	10	46	15	50	11	50	11
Female (n = 9)	2	7	2	7	6	3	8	1	5	4	9	0
Age												
<65 (n = 26)	6	20	6	20	21	5	21	5	20	6	20	6
≥65 (n = 44)	12	32	4	40	36	8	33	11	35	9	39	5
Tumor size^a^												
<3 cm (n = 35)	10	25	3	32	29	6	27	8	28	7	30	5
≥3 cm (n = 35)	8	27	7	28	28	7	27	8	27	8	29	6
Multiplicity												
Solitary (n = 34)	10	24	4	30	30	4	28	6	26	8	28	6
Multiple (n = 36)	8	28	6	30	27	9	26	10	29	7	31	5
Depth of invasion												
pT1‐T2 (n = 41)	8	33	4	37	31	10	26	15**	27	14**	30	11**
pT3‐T4 (n = 29)	10	19	6	23	26	3	28	1	28	1	29	0
Histologic grade												
Low (n = 30)	5	25	3	27	25	5	19	11*	17	13***	22	8*
High (n = 40)	13	27	7	33	32	8	35	5	38	2	37	3
Lymphatic met.												
No (n = 40)	6	34*	7	33	29	11*	27	13*	27	13*	30	10*
Yes (n = 30)	12	18	3	27	28	2	27	3	28	2	29	1

Abbreviations: Circ‐1946, hsa_circ_0001946. Exp., Expression level; Circ‐3141, hsa_circ_0003141; Circ‐4826, hsa_circ_0004826; Circ‐6782, hsa_circ_0026782; Circ‐7837, hsa_circ_0077837; Circ‐8039, hsa_circ_0008039; Lymphatic met., Lymphatic metastasis.

^a^ The patients were distinguished into two groups according to the long diameter of tumor in the median that is 3 cm.

* *P* < .05;

** *P* < .01;

*** *P* < .001; Fisherʹs exact test.

**FIGURE 7:**
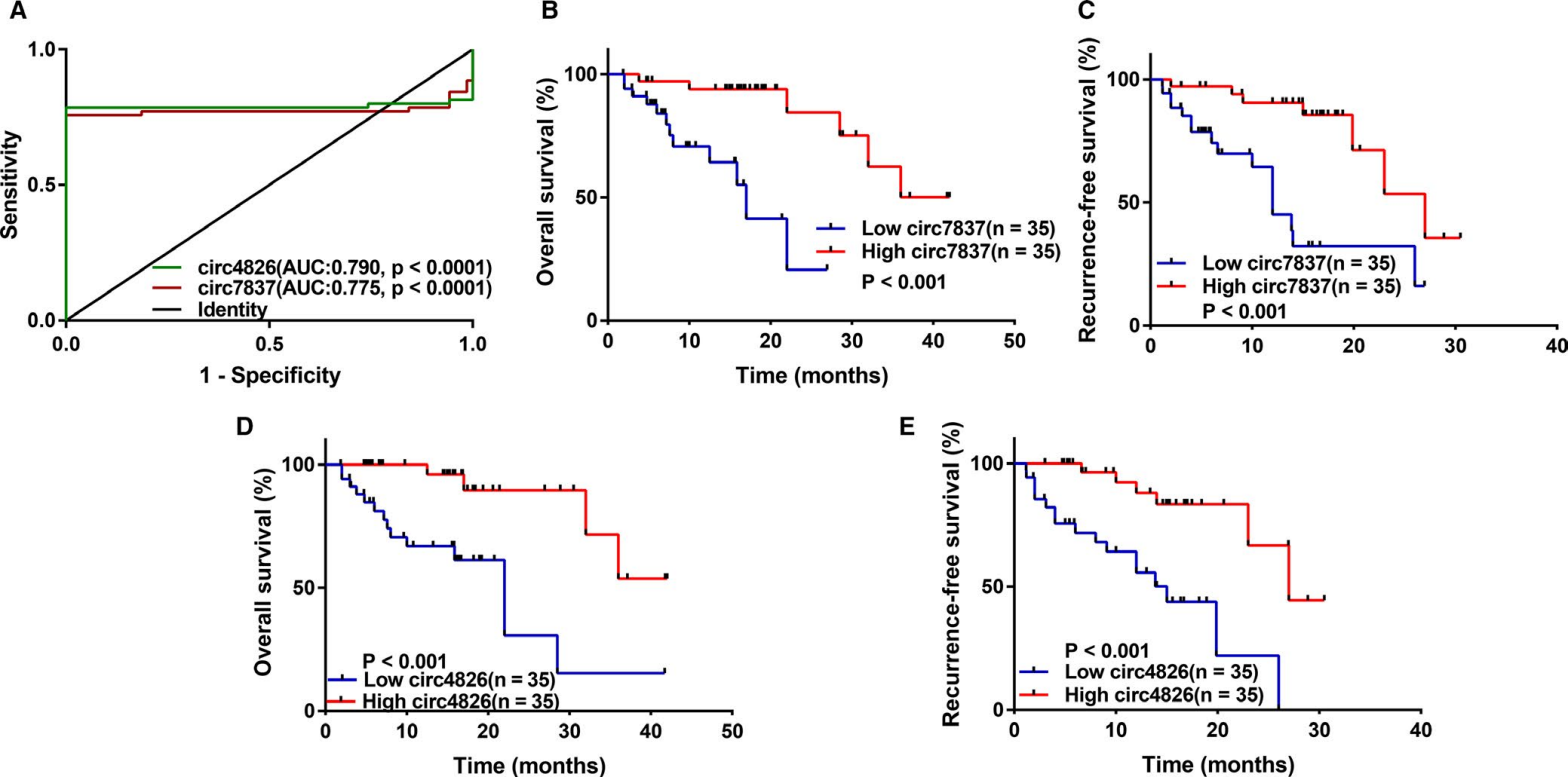
The circ_0004826 and circ_0077837, best emerging as ideal biomarkers for clinical prognosis and diagnosis. (A) The receiver operating characteristic (ROC) curve has been used to evaluate circ_0004826 and circ_0077837 potential diagnostic value. The area under the ROC curve (AUC) of them was 0.790 and 0.775, respectively. Circ‐4826, hsa_circ_0004826; Circ‐7837, hsa_circ_0077837. (B and C) Kaplan‐Meier plots with univariate analyses of overall survival (OS) and recurrence‐free survival (RFS) in 70 patients with BC according to circ_0077837 expression levels. Low level of circ_0077837 is correlated with reduced OS and RFS. (D and E) Kaplan‐Meier plots with univariate analyses of OS and PFS in 70 patients with BC according to circ_0004826 expression levels. Low level of circ_0004826 is associated with poor OS and RFS

Furthermore, we performed the receiver operator characteristic (ROC) curve analysis to research the diagnostic value of circ_0077837 and circ_0004826 in distinguishing BC samples from adjacent noncancerous samples. When the expression level of circ_0077837 and circ_0004826 was analyzed for this purpose, the area under the ROC curve (AUC) was 0.775 (95% CI 0.68‐0.87, *P* < .0001), 0.790 (95% CI 0.70‐0.88, *P* < .0001), respectively (Figure 7A). Consistently, Kaplan‐Meier plots manifested that lower expression of circ_0077837 and circ_0004826 was correlated with shorter overall survival (OS) and recurrence‐free survival (RFS) time of patients with BC (Figure 7B‐E). Moreover, Cox regression analysis also confirmed the independent prognostic value of circ_0077837, circ_0004826, and lymphatic metastasis in BC patients (Table 4). Together, these results disclosed a link between downregulation of hsa_circ_0077837, hsa_circ_0004826, and aggressive Clinicopathological characteristics, adverse prognosis of BC might act as a vital role in carcinogenesis and progression of BC patients.

**TABLE 4:** Univariate and multivariate analysis of prognostic factors for OS and RFS in BC patients

Factors	Univariate analysis (OS)		Multivariate analysis (OS)		Univariate analysis (RFS)		Multivariate analysis (RFS)	
	HR (95% CI)	*P*‐value	HR (95% CI)	*P*‐value	HR (95% CI)	*P*‐value	HR (95% CI)	*P*‐value
Age (≥65/<65)	1.687 (0.59‐4.84)	.33	–	–	1.444 (0.51‐4.12)	.493	–	–
Gender (male/female)	2.341 (0.31‐17.80)	.411	–	–	2.622 (0.34‐20.12)	.354	–	–
Tumor size (≥3 cm/< 3cm)	1.262 (0.48‐3.33)	.639	–	–	1.341 (0.51‐3.55)	.555	–	–
Mul. (multiple/single)	1.545 (0.80‐2.99)	.196	–	–	1.189 (0.41‐3.42)	.749	–	–
Lymph no. Met. (Yes/No)	9.099 (2.59‐32.00)	.001^a^	3.427 (1.14‐10.33)	.029^a^	15.472 (3.44‐69.52)	<.001^a^	11.114 (1.19‐103)	.035^a^
T stage (T2‐T4/Ta‐T1)	7.666 (2.18‐26.93)	.001^a^	1.112 (0.32‐3.86)	.868	13.923 (2.97‐65.18)	.001^a^	1.702 (0.20‐14.7)	.628
Grade (high/low)	6.394 (1.77‐23.06)	.005^a^	1.634 (0.44‐6.13)	.467	9.48 (2.08‐43.27)	.004^a^	0.874 (0.10‐7.91)	.905
Circ7837 (high/low)	0.130 (0.03‐0.5)	.003^a^	0.352 (0.15‐0.82)	.016^a^	0.192 (0.06‐0.63)	.006^a^	0.232 (0.06‐0.97)	.045^a^
Circ4826 (high/low)	5.706 (1.81‐18.03)	.003^a^	0.352 (0.16‐0.77)	.008^a^	0.190 (0.06‐0.60)	.004^a^	0.252 (0.06‐1.07)	.062

Abbreviations: Circ4826, hsa_circ_0004826; Circ7837, hsa_circ_0077837; Lymph no. Met., Lymph node metastasis; Mul., Multiplicity; OS, Overall survival; RFS: Recurrence‐free survival.

^a^ Indicated statistical significance.

### 3.6 The biological function of hsa_circ_0077837 and hsa_circ_0004826 in vitro

**FIGURE 8:**
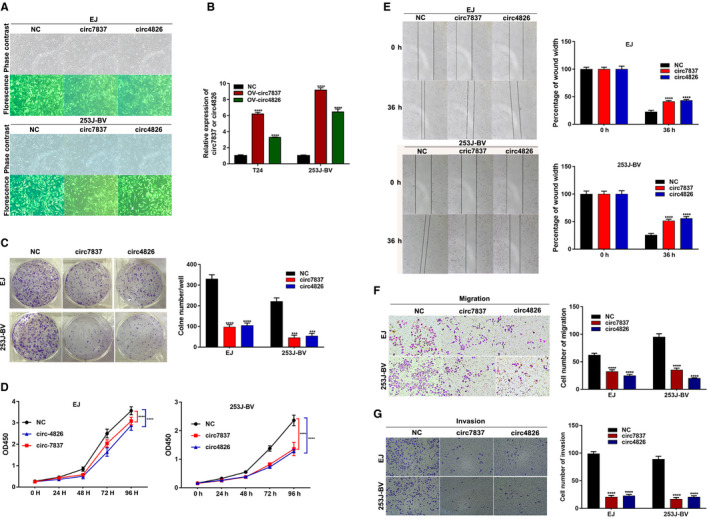
Both circ_0077837 and circ_0004826 exerted a tumor suppressive effect in BC cells. A, Transgene expression in EJ and 253J‐BV bladder cancer cell lines transduced with lentiviral vectors. Upper panels show phase contrast photomicrograph and lower panels show GFP fluorescence of the same field. B, Overexpression of circ_0077837 and circ_0004826 was confirmed via qRT‐PCR in BC cell lines EJ and 253J‐BV. C, Cell colony formation numbers were counted after transfection with OV‐NC, OV‐circ_7837, or OV‐circ_4826. D, CCK‐8 assays were utilized to detect cell proliferation ability in EJ and 253J‐BV cells transfected with OV‐NC, OV‐circ_7837, or OV‐circ_4826. E and F, The migration potential of EJ and 253J‐BV cells transfected with the OV‐circ_7837 or OV‐circ_4826 than that treated with OV‐NC was damaged by the wound healing assay at 36 h after scratch and transwell migration assay (without Matrigel) at 36 h after incubation. G, Overexpression of OV‐circ_7837 or OV‐circ_4826 all impaired the invasive capacity of EJ and 253J‐BV cells as detected by transwell Matrigel invasion assay. Data are presented as means ± SD; n = 3. SD: standard deviation. OV‐: Overexpression; NC, empty vector; Circ‐7837, hsa_circ_0077837; Circ‐4826, hsa_circ_0004826; CCK‐8: cell counting kit‐8; OD: optical density. *P < .05, **P < .01 and ***P < .001

Given the expression level of circ_0077837 (circ_7837) and circ_0004826 (circ_4826) in the previously presented four BC cell lines, EJ and 253J‐BV with the varying degrees of downregulated expression were chosen as the experimental candidates to further explore functional role of this two circRNAs (Figure 4C). Then, to explore the role circ_7837 and circ_4826 plays in human bladder cancer, EJ and 253J‐BV cells were transduced using the lentiviral vector with OV‐circ_7837, OV‐circ_4826 or corresponding NC. GFP fluorescence was used as a reporter gene. The high rate of GFP‐positive cells was observed by inverted fluorescence microscopy at 72h postinfection (Figure 8A), which suggests the high efficiency of infection.

After 72h, circ_0004826 and circ_0077837 expression was effectively increased in cells transfected with OV‐circ_7837 and OV‐circ_4826, respectively, relative to the cells transfected with NC (Figure 8B). The CCK‐8 and clone formation experiment results showed that the overexpression treatment markedly weakened the proliferation ability of EJ and 253J‐BV cell lines compared with NC (Figure 8C,D). Next, we investigated the impacts of circ_0077837 and circ_0004826 on BC cells migration and invasion capacity. Wound healing assay revealed that circ_0077837 or circ_0004826 overexpression dramatically decreased BC cells migration ability compared with NC group (Figure 8E). Consistently, transwell assay with or without Matrigel indicated that circ_0077837 overexpression excessively inhibited the migration and invasion of EJ and 253J‐BV cell lines. Ectopic circ_0004826 expression also showed the similar results (Figure 8F,G). Taking together, these results suggested that circ_0077837 and circ_0004826 played a crucial role in BC cell proliferation, migration, and invasion, which was well consistent with previous clinical results.

## 4 DISCUSSION

Bladder cancer (BC) remains to be a malignant tumor with unsatisfactory therapeutic effects in clinical practice. So it is an urgent problem to investigate the molecular mechanism participated in BC progression. Recently, a mounting number of works have elucidated that circRNAs play significant roles in the carcinogenesis and cancer progression. For example, it have been found that Circ‐UBAP2 plays a important role in osteosarcoma development^39^ and the proliferation and invasion of human lung cancer.^40^ Li et al CircDDX17 functions as a tumor suppressor and could act as a potential biomarker and a therapeutic target for colorectal cancer.^26^ In the bladder cancer, Zheng et al describe circHIPK3 which effectively inhibits migration, invasion, and angiogenesis of BC cells by miR‐558/heparanase (HPSE) pathway.^41^ However, although numerous circRNAs have been annotated in recent years, reports on global circRNAs profiles remain largely unknown, especially in BC. Therefore, we performed a RNA‐seq analysis to acquire the whole transcripts expression profile in BC. More importantly, we authenticated two novel circRNA biomarkers for BC clinical diagnosis and patientsʹ survival evaluation.

In our research, we successfully identified a set of dysregulated circRNAs and mRNAs in BC tissues compared with paired adjacent normal tissues using high‐throughput sequencing. Among them, 40 and 47 circRNAs were obviously upregulated and downregulated in BC, respectively. Analogously, 1467 upregulated and 1289 downregulated mRNAs were determined. Of them, Liu Y et al reported that circular RNA hsa_circ_0008039 promotes breast cancer cell proliferation and migration by modulating miR‐432‐5p/E2F3 axis and hence may be a potential therapeutic target.^42^ Huang et al argued that hsa_circ_000074, as a tumor promoter in CC, enhances the cellʹs ability to proliferate, migrate, and invade by reducing the expression of E‐cad and is a candidate target for the therapy of CC in the clinic.^43^ It has been confirmed that hsa_circ_0001946 has been also reported to be extremely downregulated in ESCC tissues by Fanʹs teams^44^ and in NSCLC samples by Huangʹs group^45^ using another microarray platform and RNA sequencing technology, which confirms the accuracy of our microarray result and potentially implies the crucial role of circ_0001946 in the development of BC as well.

CircRNAs can regulate the transcription or translation of parent genes.^36^, ^37^, ^46^, ^47^ In this study, the possible GO functional terms and signaling pathways of the host genes of these DE circRNAs were characterized. The results showed that these circRNAs are highly enriched in a few crucial pathways related with cancer, such as the “Regulation of actin cytoskeleton, Focal adhesion, Pyruvate metabolism, Bacterial invasion of epithelial cells, ECM‐receptor interaction,” etc, further revealing the potential biofunctional roles for circRNAs as triggers of BC. Prior studies have found that the activation of the actin cytoskeleton promote bladder cancer cell growth, proliferation, and metastasis.^48^, ^49^ Yamasaki T et al suggested that the loss of tumor suppressive miR‐218 enhances cancer renal cell migration and invasion through dysregulation of the focal adhesion pathway.^50^ And thoughtfully, the host genes of these deregulated circRNAs were distributed across all chromosomes, manifesting that circRNAs are extensively involved in the modulation of many genes. However, as to whether circRNAs can regulate parental genes, we need to do some molecular mechanism experiments in the future research plans.

Next, to further screen functional circRNA, we first analyzed the linear counterparts of these circRNAs in line with the strategy adopted in the previous study,^51^ and eight circRNAs attracted our attention. The circ_0026782, circ_0001946, circ_0004826, circ_0077837, circ_0030586, circ_0008039, circ_0001346, and circ_0003141 are spliced from ITGA7, CDR1, UTRN, EPB41L2, ABCC4, PRKAR1B, RNF13, and UBAP2, which all play an important effect in tumor proliferation, migration, and metastasis. Bhandari A et al reported that ITGA7 acts as a tumor suppressor and regulates migration and invasion in breast cancer.^52^ According to cell proliferation assays as well as the mouse xenograft model, Li et al determined UTRN as a breast cancer suppressor gene both in vitro and in vivo.^53^ In ovarian cancer (OC), Seborova et al^54^ indicated that downregulation of ABCC4, called the “Multidrug resistance protein 4,” was associated with the best sensitivity to chemotherapy and time to progression. The previous studies has shown that the overexpression of a wild‐type RNF13 in murine melanoma cell line (B16F10) restrained the colonization of tumor cells in the lung.^55^

CircRNAs play important pathological and physiological functions in diverse ways, for instance, functioning as miRNA sponges,^5^, ^47^ binding to RNA‐binding proteins (RBPs) or other functional proteins,^56^ modulating alternative splicing or the expression of parental gene,^37^ regulating protein translation and even translating into proteins.^15^, ^57^ Among these roles of circRNA, acting as a miRNA sponge represents the most common function. Previous plenty of findings have established that circRNAs can serve as sponges to miRNAs to affect cancer cell proliferation and invasion.^20^, ^38^, ^58^

Herein, according to strict bioinformatics analysis, we further obtained the five key miRNAs that potentially combine with each of the selected eight circRNAs, and predicted total 94 miRNAs‐targeted critical DE‐genes derived from our mRNAs sequencing data and constructed an interaction ceRNA network of them (see Figure 5B). In the same way, we further established ceRNA network associated with entire DE‐circRNAs (see Figure 5A). The ceRNA network exhibits considerable clues for comprehending the key effects of ceRNA‐mediated gene modulatory networks in BC genesis and evolution. Interestingly, as shown in Figure 5, a few miRNAs, including hsa‐miR‐558, hsa‐miR‐7, hsa‐miR‐203, hsa‐miR‐145, hsa‐miR‐100, hsa‐miR‐217, etc, has been proven to be a tumor promoter or suppressor in bladder carcinoma.^41^, ^59^, ^60^, ^61^, ^62^, ^63^ This report further supported the hypothesis that these circRNAs act as a miRNA harbor in bladder cancer.

To further explore the roles of these all DE‐circRNAs on downstream genes, GO enrichment, KEGG pathway analysis were also conducted to evaluate the predicted critical genes of them. As shown in Figure 3B,C, several cancer‐related pathways, such as pathways in cancer, cell cycle, PI3K‐Akt signaling pathway, MAPK signaling pathway, cellular senescence, were significantly enriched and promote various cancer cell growth, invasion, and metastasis.^56^, ^64^, ^65^ Furthermore, protein‐protein interaction (PPI) network and module analysis of 94 DE‐genes predicted by screened eight DE circRNAs are delineated in Figure 6. As for the first two DE‐genes modules in the PPI network, they was associated with the pathway of “cell cycle” and “pathways in cancer”, respectively. For the two genes modules, CCNB1 and EGF was the top nodes involved in the most protein‐protein pairs, respectively. Hence, we speculate that circRNAs may promote BC progression by harboring miRNAs and regulating these pathways‐correlated genes. Nevertheless, future studies are needed to illustrate the specific cellular process in which these circRNAs involves.

In the next step, we further tested the eight circRNAs in 70 pairs of BC and adjacent normal tissues, and the qRT‐PCR data are approximately consistent with the sequencing results, which demonstrated that the sequencing results are credible. For the first time, we performed correlation analysis between these circRNAs expression and several clinical features of BC in detail based on the above qRT‐PCR validation results. We found that low circ_0026782, circ_0077837, circ_0004826, and circ_0001946 as well as high circ_0003141 expression level were closely connected with various poorer clinicopathological characteristics of BC, including greater tumor depth, lymph node metastasis, and advanced T stages.

Hence, these circ_0077837 and circ_0004826 circRNAs deserve further exploration in the diagnosis and treatment of BC. To probe into the significance of these circRNAs in distinguishing BC tissues and adjacent normal tissues, ROC curves was drawn, and the AUC of circ_0077837 and circ_0004826 were 0.775 (*P* < .0001) and 0.790 (*P* < .0001), respectively.

Furthermore, prior works suggested that circRNAs act as prognostic biomarkers for malignancies, for example, circTADA2As and circEPSTI1 for breast cancer,^66^, ^67^ Hsa_circ_0003998 for hepatocellular carcinoma,^68^ circEXOC6B and circN4BP2L2 for epithelial ovarian cancer,^69^ Hsa_circ_0001946 for ESCC,^44^ and circRNA_0001178 and circRNA_0000826 for colorectal cancer.^70^ Similar to these reports, our study revealed that the OS and RFS survival rate of BC patients with low circ_0077837 and circ_0004826 expression had a significantly lower compared to those with high circ_0077837 and circ_0004826 expression using Kaplan‐Meier plot analysis. And more notably, univariate and multivariate Cox model analysis further elucidated that lower expression of circ_0077837 and circ_0004826 equally stood out as a potential independent prognostic factor for both poor OS and RFS of BC patients. All these stated earlier manifested that circ_0077837 and circ_0004826 may serve as significant and useful biomarkers for BC diagnosis and prognosis.

Next, in order to understand the biological functions of circ_0077837 and circ_0004826, we examined the biological roles by overexpressing circ_0077837 or circ_0004826 among EJ and 253J‐BV cells. CCK‐8 assay and clone formation assay demonstrated that the overexpression circ_0077837 or circ_0004826 significantly decreased BC cells growth. And that, we explored the impacts of circ_0077837 and circ_0004826 on BC cells migration and invasion. Wound healing assay and transwell assay exhibited that ectopic expression circ_0077837 or circ_0004826 dramatically restrained BC cells migration and invasion capacity. Therefore, these data showed that both circ_0077837 and circ_0004826 could work as a tumor suppressor in BC development and progression.

In some ways, our experiments have some limitations. The clinical significance of circ_0077837 and circ_0004826 may require to be addressed in larger cohorts. Due to time constraints, we did not verify the corresponding downstream miRNAs and proteins of circ_0077837 or circ_0004826; thus, more experiments are needed to clarify the mechanistic link between circ_0077837 or circ_0004826 and the malignant progression of BC. What is more, although tumor suppressor functions were determined, the use of circ_0077837 and circ_0004826 in clinical therapies is still in the distant future.

## 5 CONCLUSIONS

In summary, we firstly demonstrated that circ_0077837 and circ_0004826 were significantly downregulated in BC and closely correlated with ill clinicopathological characteristics. Moreover, we showed that circ_0077837 and circ_0004826 could serve as a tumor suppressor in BC cells. Accordingly, our findings highlighted that both circ_0077837 and circ_0004826 might play a crucial role in BC progression and were a promising biomarker for BC prognosis and therapy.

## CONFLICT OF INTERESTS

The authors declare that they have no competing interests.

## AUTHORS CONTRIBUTIONS

HH and CS conceived and designed the research. CS, HH, DT, and ZW performed the research and analyzed results. CS wrote the manuscript. YW, SG, LD, LX, and YQ edited the manuscript and provided critical comments. HH provided the financial support and supervised laboratorial processes. All authors read and approved the final manuscript.

## Supporting information

Fig S1

Fig S2

Fig S3

Table S1‐S3

## Data Availability

The datasets used and/or analyzed during this study are available from the corresponding author on reasonable request.
