# Supplementary material for: Downregulated hsa_circ_0077837 and hsa_circ_0004826, facilitate bladder cancer progression and predict poor prognosis for bladder cancer patients
Source: Cancer Med. 2020 Apr 6;9(11):3885–903. doi: 10.1002/cam4.3006 (PMC7286451; doi:10.1002/cam4.3006)
Supplement: Supplementary file 1 — Fig S1 [file CAM4-9-3885-s001.pdf]

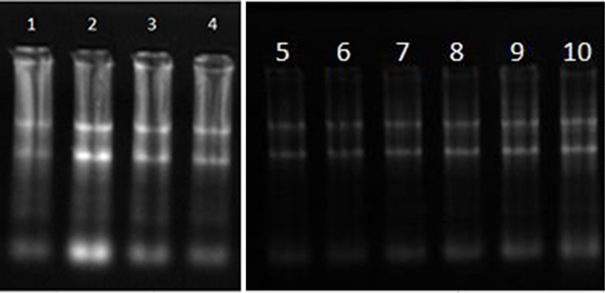

Lane 1: C8.Input Lane 2: N8.Input Lane 3: C12.Input Lane 4: N12.Input

Lane 5: C19.Input Lane 6: N19.Input Lane 7: C25.Input Lane 8: N25.Input

Lane 9: C37.Input Lane 10: N37.Input
