# Supplementary figures and images for: Downregulated hsa_circ_0077837 and hsa_circ_0004826, facilitate bladder cancer progression and predict poor prognosis for bladder cancer patients
Source: Cancer Med. 2020 Apr 6;9(11):3885–903. doi: 10.1002/cam4.3006 (PMC7286451; doi:10.1002/cam4.3006)

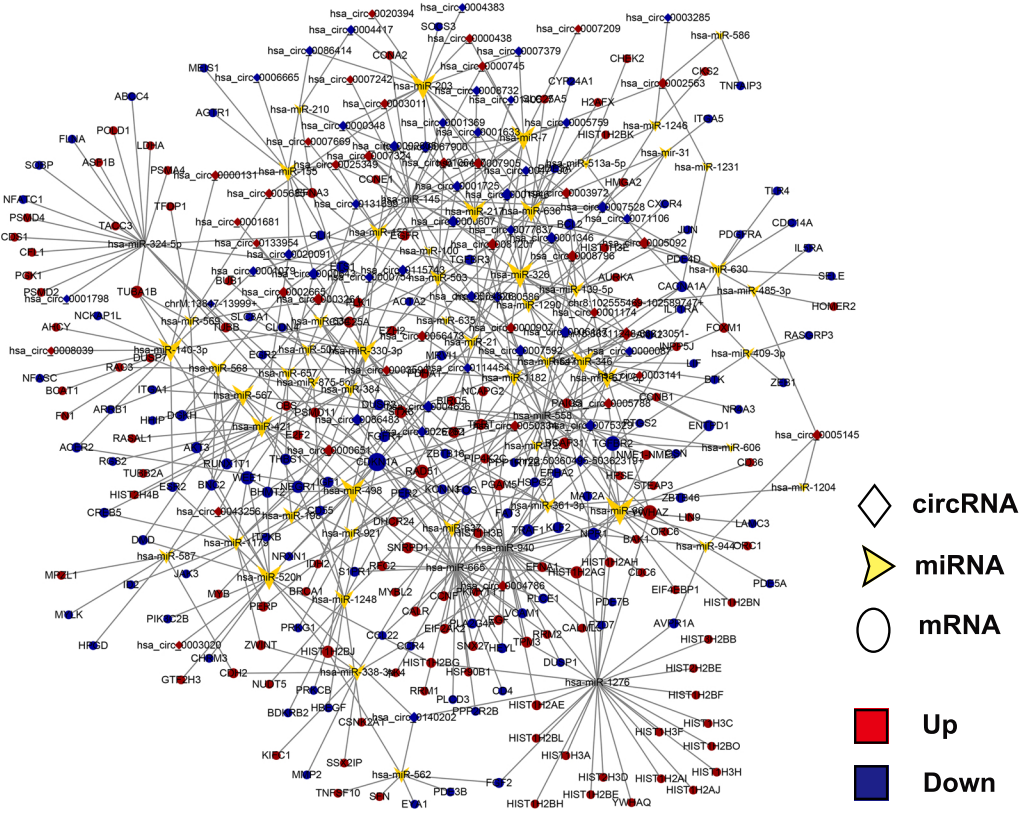

Supplement: Supplementary file 2 — Fig S2 [file CAM4-9-3885-s002.pdf]
