# Supplementary material for: Downregulated hsa_circ_0077837 and hsa_circ_0004826, facilitate bladder cancer progression and predict poor prognosis for bladder cancer patients
Source: Cancer Med. 2020 Apr 6;9(11):3885–903. doi: 10.1002/cam4.3006 (PMC7286451; doi:10.1002/cam4.3006)
Supplement: Supplementary file 3 — Fig S3 [file CAM4-9-3885-s003.pdf]

A

<table>
<tr><th>CircRNA Mirbase ID</th><th>CircRNA (Top) miRNA (Bottom) pairing</th><th>Site Type</th><th>CircRNA Start</th><th>CircRNA End</th></tr>
<tr><td>hsa_circ_0077837 (5' ... 3')
hsa-miR-7 (3' ... 5')</td><td>AAAUCAGCAGAAUCA---GUCUUCCG
||||| |||||||
UGUUGUUUUAGUGAUCAGAAGGU</td><td>7mer-m8</td><td>125</td><td>131</td></tr>
<tr><td>hsa_circ_0077837 (5' ... 3')
hsa-miR-21 (3' ... 5')</td><td>NNNNNNNNNNNNNNNCAUAAGCUG
|||||||
AGUUGUAGUCAGACUAUUCGAU</td><td>7mer-m8</td><td>2</td><td>8</td></tr>
<tr><td>hsa_circ_0077837 (5' ... 3')
hsa-miR-942 (3' ... 5')</td><td>ACGCCGCCAGAAGAGAGAGAAGG
|||| |||||||
GUGUACCGGUUUUG--UCUCUUCU</td><td>7mer-m8</td><td>206</td><td>212</td></tr>
<tr><td>hsa_circ_0077837 (5' ... 3')
hsa-miR-942 (3' ... 5')</td><td>UUAGUAAGUAAGGAGAGAGAAGA
|||||||
GUGUACCGGUUUUGUCUCUUCU</td><td>8mer-1a</td><td>531</td><td>538</td></tr>
<tr><td>hsa_circ_0077837 (5' ... 3')
hsa-miR-942 (3' ... 5')</td><td>AAGUAAGGAGAGAGAAGAGAAGG
|||||||
GUGUACCGGUUUUGUCUCUUCU</td><td>7mer-m8</td><td>536</td><td>542</td></tr>
<tr><td>hsa_circ_0077837 (5' ... 3')
hsa-miR-942 (3' ... 5')</td><td>CAAUGAGCUGAAAGCAGAGAAGG
|||||||
GUGUACCGGUUUUGUCUCUUCU</td><td>7mer-m8</td><td>626</td><td>632</td></tr>
<tr><td>hsa_circ_0077837 (5' ... 3')
hsa-miR-1205 (3' ... 5')</td><td>GGUUCCCAGCCACCUCCUGCAGC
||| |||||||
GAGUUUCGUUUG-GGACGUCU</td><td>7mer-m8</td><td>165</td><td>171</td></tr>
<tr><td>hsa_circ_0077837 (5' ... 3')
hsa-miR-1236 (3' ... 5')</td><td>UAGUCUACGCCGCCAGAAGAGAG
||||||
GACCUCUCUGUUCCCCUUCUCC</td><td>7mer-1a</td><td>200</td><td>206</td></tr>
<tr><td>hsa_circ_0077837 (5' ... 3')
hsa-miR-1236 (3' ... 5')</td><td>GUAAGUAAGGAGAGA------GAAGAGAA
||||||| ||||||
GACCUCUCUGUUCCCCUUCUCC</td><td>7mer-1a</td><td>534</td><td>540</td></tr>
</table>

B

<table>
<tr><th>CircRNA Mirbase ID</th><th>CircRNA (Top) miRNA (Bottom) pairing</th><th>Site Type</th><th>CircRNA Start</th><th>CircRNA End</th></tr>
<tr><td>hsa_circ_0004826 (5' ... 3')
hsa-miR-145 (3' ... 5')</td><td>AUAUAAUCAGUGAGAAACUGGAG
|||||||
UCCCUAAGGACCCUUUUGACCUG</td><td>7mer-m8</td><td>107</td><td>113</td></tr>
<tr><td>hsa_circ_0004826 (5' ... 3')
hsa-miR-558 (3' ... 5')</td><td>GGGGAGCUGGACAAACAGCUCAC
||| ||||||
UAAAACCAUGUCGUCGAGU</td><td>7mer-1a</td><td>253</td><td>259</td></tr>
<tr><td>hsa_circ_0004826 (5' ... 3')
hsa-miR-326 (3' ... 5')</td><td>UCAGCCCCUGACCUCCCCAGAGA
|||||||
GACCUCCUUCCCGGGUCUCC</td><td>8mer-1a</td><td>393</td><td>400</td></tr>
<tr><td>hsa_circ_0004826 (5' ... 3')
hsa-miR-330-5p (3' ... 5')</td><td>UCAGCCCCUGACCUC--CCCAGAGA
||| |||||||
CGGAUUCUGUGUCCGGGUCUCU</td><td>8mer-1a</td><td>393</td><td>400</td></tr>
<tr><td>hsa_circ_0004826 (5' ... 3')
hsa-miR-1282 (3' ... 5')</td><td>UAUUGUCCAGAAACA-GCAAACGG
|||||| |||||||
UUCGUCUUUUUCCGUUUGCU</td><td>7mer-m8</td><td>696</td><td>702</td></tr>
</table>
