# Supplementary material for: Downregulated hsa_circ_0077837 and hsa_circ_0004826, facilitate bladder cancer progression and predict poor prognosis for bladder cancer patients
Source: Cancer Med. 2020 Apr 6;9(11):3885–903. doi: 10.1002/cam4.3006 (PMC7286451; doi:10.1002/cam4.3006)
Supplement: Supplementary file 4 — Table S1‐S3 [file CAM4-9-3885-s004.doc]

**Supplementary files**

**Circular RNA profiling and functional tests reveal circ_0077837 and circ_0004826 inhibit growth and invasion and serve as prognostic biomarkers in bladder cancer**

**Results**

**Table S1.** Data statistics of patients with BC.

| **Variables** | **No. of patients (%)** |
| --- | --- |
| Gender |  |
| Male | 61 (87.1%) |
| Female | 9 (12.9%) |
| Age |  |
| ＜60 | 18(25.7%) |
| ≥60 | 52(74.3%) |
| Tumor size |  |
| ＜3cm | 35(50%) |
| ≥3cm | 35(50%) |
| Number of tumors |  |
| Solitary | 32(45.7%) |
| Multiple | 38(54.3%) |
| Pathology stage |  |
| pTa-pT1 | 32(45.7%) |
| pT2-T4 | 38(54.3%) |
| Histologic grade |  |
| Low | 28(40%) |
| High | 42(60%) |
| Lymphatic metastasis |  |
| Yes | 40(57.1%) |
| No | 30(42.9%) |

**Table S2.** Screening for bladder cancer-related DE circRNAs by circRNA sequencing analysis.

**A**, Upregulated circRNAs in the bladder cancer samples compared with adjacent normal samples.

| **Chrom** | **Genesymbol** | **circBaseID** | **logFC** | **PValue** |
| --- | --- | --- | --- | --- |
| chr7:22347958-22357656- | RAPGEF5 | hsa_circ_0133954 | 4.83 | 0.00335 |
| chr7:22330794-22357656- | RAPGEF5 | hsa_circ_0001681 | 4.54 | 0.00517 |
| chr15:86311248-86313051- | KLHL25 | * | 4.50 | 0.00763 |
| chr12:7556131-7586290- | CD163L1 | hsa_circ_0025349 | 4.22 | 0.01248 |
| chr12:111951161-111957880- | ATXN2 | hsa_circ_0000438 | 4.15 | 0.01625 |
| chr1:151611364-151641111+ | SNX27 | hsa_circ_0000131 | 4.07 | 0.01799 |
| chr17:35604935-35609962- | ACACA | hsa_circ_0043256 | 4.03 | 0.01865 |
| chr7:97820926-97823884+ | LMTK2 | hsa_circ_0081207 | 3.98 | 0.01242 |
| chr1:62321702-62350080+ | INADL | hsa_circ_0012779 | 3.86 | 0.02621 |
| chr9:22046750-22097363+ | CDKN2B-AS1 | hsa_circ_0008796 | 3.80 | 0.02795 |
| chr5:14673751-14681716+ | OTULIN | hsa_circ_0005145 | 3.79 | 0.02763 |
| chr15:78837211-78838106+ | PSMA4 | hsa_circ_0003590 | 3.78 | 0.03261 |
| chr10:51606988-51613311- | TIMM23 | hsa_circ_0003020 | 3.77 | 0.03167 |
| chr9:33953283-33973235- | UBAP2 | hsa_circ_0003141 | 3.73 | 0.03985 |
| chr8:102555469-102589747+ | GRHL2 | * | 3.73 | 0.03370 |
| chr15:90982564-90986710+ | IQGAP1 | hsa_circ_0000651 | 3.73 | 0.03287 |
| chr17:27613010-27614734- | NUFIP2 | hsa_circ_0106467 | 3.72 | 0.03421 |
| chr20:58755108-58755971+ | RP5-1043L13.1 | hsa_circ_0001174 | 3.72 | 0.03157 |
| chr10:128768966-128860040+ | DOCK1 | hsa_circ_0020394 | 3.72 | 0.03137 |
| chr9:96238538-96261168+ | FAM120A | hsa_circ_0003972 | 3.71 | 0.03854 |
| chr2:160964190-160983112- | ITGB6 | hsa_circ_0056854 | 3.69 | 0.04646 |
| chr2:131796426-131801156+ | ARHGEF4 | hsa_circ_0056473 | 3.63 | 0.03796 |
| chr1:176132005-176145143- | RFWD2 | hsa_circ_0003011 | 3.58 | 0.03983 |
| chr1:39900086-39905172+ | MACF1 | hsa_circ_0005788 | 3.55 | 0.04540 |
| chr19:46024580-46026076+ | VASP | hsa_circ_0004786 | 3.55 | 0.04534 |
| chr8:135612679-135622898- | ZFAT | hsa_circ_0007209 | 3.53 | 0.04016 |
| chr1:45223228-45223812+ | KIF2C | hsa_circ_0002563 | 3.50 | 0.04767 |
| chr11:9451221-9452550+ | IPO7 | hsa_circ_0005092 | 3.50 | 0.03096 |
| chr19:30462100-30477324+ | URI1 | hsa_circ_0050334 | 3.47 | 0.04826 |
| chr10:13169745-13178897+ | OPTN | hsa_circ_0007242 | 3.40 | 0.04286 |
| chr16:68308594-68309152+ | SLC7A6 | hsa_circ_0007669 | 3.28 | 0.04905 |
| chr4:87693931-87696805+ | PTPN13 | hsa_circ_0007324 | 3.23 | 0.02906 |
| chr18:33775220-33783152+ | MOCOS | hsa_circ_0003552 | 3.22 | 0.03144 |
| chr7:716866-751164- | PRKAR1B | hsa_circ_0008039 | 3.03 | 0.03494 |
| chr7:72361166-72361649+ | POM121 | hsa_circ_0003261 | 2.87 | 0.04738 |
| chr19:17212470-17213367+ | MYO9B | hsa_circ_0000907 | 2.79 | 0.01162 |
| chr1:180953813-180962561- | STX6 | hsa_circ_0007905 | 2.48 | 0.01962 |
| chr10:5836848-5842668- | GDI2 | hsa_circ_0002665 | 2.48 | 0.04911 |
| chr17:20107646-20109225+ | SPECC1 | hsa_circ_0000745 | 2.33 | 0.04154 |
| chr3:149563798-149639014+ | RNF13 | hsa_circ_0001346 | 2.13 | 0.03256 |

*indicated novel circRNAs.

**B**, Downregulated circRNAs in the bladder cancer samples compared with adjacent normal samples.

| **Chrom** | **Genesymbol** | **circBaseID** | **logFC** | **PValue** |
| --- | --- | --- | --- | --- |
| chr11:92085262-92088570+ | FAT3 | hsa_circ_0000348 | -5.80 | 0.00027 |
| chr12:56094683-56094938- | ITGA7 | hsa_circ_0026782 | -4.95 | 0.00015 |
| chr6:131247745-131277639- | EPB41L2 | hsa_circ_0077837 | -4.88 | 0.00020 |
| chrM:13847-13999+ | MTND5 | * | -4.74 | 0.00698 |
| chr15:62299507-62306191- | VPS13C | hsa_circ_0000607 | -4.54 | 0.01057 |
| chr12:2364966-2365205+ | CACNA1C | * | -4.27 | 0.01824 |
| chr6:37617885-37620116- | MDGA1 | hsa_circ_0131699 | -4.21 | 0.02302 |
| chr10:15875629-15889942- | FAM188A | hsa_circ_0006665 | -4.20 | 0.02093 |
| chr1:219366424-219414650+ | LYPLAL1 | hsa_circ_0004417 | -4.19 | 0.02302 |
| chr5:179315103-179315312- | TBC1D9B | hsa_circ_0075329 | -4.17 | 0.02223 |
| chr6:144808684-144814592+ | UTRN | hsa_circ_0004826 | -4.16 | 0.02355 |
| chr6:107824861-107827631+ | SOBP | hsa_circ_0001633 | -4.11 | 0.02393 |
| chr9:19423860-19435082+ | ACER2 | hsa_circ_0086483 | -4.07 | 0.03125 |
| chr7:131113792-131130662+ | MKLN1 | hsa_circ_0082415 | -4.03 | 0.02941 |
| chr10:116719485-116734144+ | TRUB1 | hsa_circ_0020091 | -3.99 | 0.02861 |
| chrX:32305646-32328393- | DMD | hsa_circ_0140202 | -3.99 | 0.02655 |
| chr18:59739906-59763183- | PIGN | hsa_circ_0007528 | -3.97 | 0.02968 |
| chr17:28011581-28030080- | SSH2 | hsa_circ_0000754 | -3.93 | 0.03342 |
| chrX:79544405-79565732- | CHMP1B2P | hsa_circ_0140637 | -3.93 | 0.01547 |
| chr9:16435553-16437522- | BNC2 | hsa_circ_0086414 | -3.92 | 0.01385 |
| chr2:169018297-169038600- | STK39 | hsa_circ_0001079 | -3.90 | 0.03899 |
| chr14:39627489-39628754- | TRAPPC6B | hsa_circ_0002395 | -3.90 | 0.03950 |
| chr13:21305980-21306260- | N6AMT2 | hsa_circ_0003285 | -3.83 | 0.04125 |
| chr4:151719233-151729550- | LRBA | hsa_circ_0004636 | -3.80 | 0.04343 |
| chr1:78183552-78191447- | USP33 | hsa_circ_0000087 | -3.80 | 0.04545 |
| chr20:35812583-35812776+ | RPN2 | hsa_circ_0005759 | -3.74 | 0.04696 |
| chr18:48444480-48458730+ | ME2 | hsa_circ_0047700 | -3.72 | 0.04883 |
| chr9:4860125-4860901+ | RCL1 | hsa_circ_0007592 | -3.71 | 0.04701 |
| chr1:92327028-92329836- | TGFBR3 | hsa_circ_0114454 | -3.67 | 0.04550 |
| chr22:50360495-50362319+ | * | * | -3.66 | 0.04225 |
| chr9:16727795-16738483- | BNC2 | hsa_circ_0008732 | -3.56 | 0.01993 |
| chr3:183368084-183369064+ | KLHL24 | hsa_circ_0001369 | -3.51 | 0.03733 |
| chr21:29816469-29818793- | AF131217.1 | hsa_circ_0115743 | -3.51 | 0.04967 |
| chr13:95813443-95840796- | ABCC4 | hsa_circ_0030586 | -3.44 | 0.04815 |
| chr7:50358644-50367353+ | IKZF1 | hsa_circ_0001708 | -3.44 | 0.03721 |
| chr20:62407031-62422143- | ZBTB46 | hsa_circ_0002805 | -3.43 | 0.03115 |
| chr5:113740135-113740553+ | KCNN2 | hsa_circ_0127664 | -3.42 | 0.03955 |
| chr8:48308936-48320523+ | SPIDR | hsa_circ_0001798 | -3.34 | 0.01862 |
| chr7:97820040-97823884+ | LMTK2 | hsa_circ_0001725 | -3.26 | 0.01814 |
| chr20:2944918-2945848+ | PTPRA | hsa_circ_0006117 | -3.25 | 0.04448 |
| chr14:35020920-35024118- | G024201 | hsa_circ_0007379 | -3.25 | 0.03886 |
| chr6:87920169-87928449+ | ZNF292 | hsa_circ_0004383 | -3.06 | 0.02273 |
| chr15:80412670-80415142+ | ZFAND6 | hsa_circ_0000643 | -2.74 | 0.04906 |
| chr3:169840379-169847340- | PHC3 | hsa_circ_0067900 | -2.64 | 0.02830 |
| chr5:95091100-95099324+ | RHOBTB3 | hsa_circ_0007444 | -2.57 | 0.02519 |
| chr4:148778704-148803083+ | ARHGAP10 | hsa_circ_0071106 | -2.32 | 0.03874 |
| chrX:139865340-139866824+ | CDR1 | hsa_circ_0001946 | -1.81 | 0.04412 |

*indicated novel circRNAs.

**Table S3. Predicted ceRNAs for circRNAs.**

| **circRNA** | **Sponge miRNA numbers** | **Target gene numbers** |
| --- | --- | --- |
| All DE-circRNAs | 59 | 227 |
| Selected eight DE-circRNAs | 16 | 94 |

DE-: differentially expressed; ceRNAs:competing endogenous RNAs.

**Figure S1.** RNA Integrity and gDNA contamination test by Denaturing Agarose Gel Electrophoresis.

**Figure S2.** The 87 dysregulated circRNAs along with 227 overlapping target mRNAs were employed to establish a ceRNAs network by Cytoscape 3.6.1 tool. The yellow arrowheads represent miRNAs. The circles represent coding genes, diamonds represent circRNAs, red indicates upregulation, and blue indicates downregulation.

**Figure S3.** circ_0077837 **(A)**, circ_0004826 **(B)** and the 5 potential key target miRNAs predicted by miRanda and CircInteractome. circRNA, circular RNA; miR, micro
